# Enpp1 deficiency caused chondrocyte apoptosis by inhibiting AMPK signaling pathway

**DOI:** 10.1186/s13018-023-03923-1

**Published:** 2023-06-27

**Authors:** Zhiqiang Gao, Qiang Wang, Kai Guo, Xinhua Li, Yufeng Huang

**Affiliations:** 1 Department of Spine Surgery, Shanghai East Hospital, School of Medicine, Tongji University, 150 Jimo Rd, Shanghai, 200092 China; 2 Department of Orthopedics, Shanghai General Hospital, Shanghai Jiao Tong University School of Medicine, Shanghai, 200080 People’s Republic of China

**Keywords:** Enpp1, Osteoarthritis, Apoptosis, AMPK, AICAR

## Abstract

**Objective and background:**

The deficiency of ectonucleotide pyrophosphatase/phosphodiesterase 1 (Enpp1) causes the phenotype similar to knee osteoarthritis (OA). However, the molecular mechanism is poorly understood.

**Method:**

The global deletion of Enpp1 (Enpp1^−/−^) mice was created to analyze the role of Enpp1 in the progress of knee OA. The apoptosis, proliferation and chondrogenic differentiation ability of chondrocytes from wild-type (WT) and Enpp1^−/−^ joints were compared. According to the results of high-throughput quantitative molecular measurements, the proteins of chondrocytes from WT and Enpp1^−/−^ mice were used to explore the mechanism of Enpp1 deficiency-associated knee OA.

**Result:**

In Enpp1^−/−^ knee joints, we found significant chondrocyte apoptosis and proteomic results showed that abnormal expression of AMP-activated protein kinase (AMPK) signaling pathway may contribute to this phenotype. In primary chondrocyte cultures in vitro, Enpp1 deletion dramatically enhancing chondrocyte apoptosis. Meanwhile, we found Enpp1 deletion inhibits the phosphorylation of AMPK (P-AMPK). We also found that decreased level of P-AMPK and chondrocyte apoptosis, which are caused by Enpp1 deficiency, can be reversed by Acadesine (AICAR), the activator of AMPK.

**Conclusion:**

Consequently, Enpp1 deficiency plays an essential role in knee OA by regulating AMPK signaling pathway.

**Supplementary Information:**

The online version contains supplementary material available at 10.1186/s13018-023-03923-1.

## Introduction

Knee OA is one of the leading causes of global disability [1] and increases the risk of all-cause mortality [2], which is further aggravating with the coming of global aging. Numerous studies have shown that the articular cartilage exhibits high apoptosis rates [3], abnormal proliferation [4] and chondrogenic differentiation [5] during the progress of knee OA.

Enpp1, a type II transmembrane glycoprotein with nucleotide pyrophosphatase and phosphodiesterase enzymatic activities [6, 7], is critical for purine metabolism, which plays an important role in cell apoptosis [8], proliferation [9] and differentiation [10]. For example, Enpp1 catalyzes the hydrolysis of adenosine triphosphate (ATP) to adenosine monophosphate (AMP) and pyrophosphate (PPi) [11].

Disorders of ATP metabolism due to Enpp1 deficiency caused OA. On the one hand, several studies have shown that articular cartilage calcification contributes to Enpp1 deficiency-associated OA of knee joints. Jin et al. [12] found that Enpp1 inhibits ectopic joint calcification and maintains articular chondrocytes by repressing hedgehog signaling pathway. Besides, Bertrand et al. [13] reported that cartilage calcification in Enpp1^−/−^ mice results in increased extracellular matrix (ECM) calcification and activation of canonical Wingless/Integrated (Wnt) signaling pathway. On the other hand, aberrant chondrocyte metabolism also plays a key role in cartilage degeneration and OA progression which is mainly regulated by the AMPK pathway [14]. Detailly, high ratio of AMP/ATP promotes the expression of P-AMPK. In joints with OA, the abnormal chondrocyte metabolism also leads to increased apoptosis, an important pathological change during OA [15]. However, it remains unclear how does the unbalanced ATP metabolism caused by Enpp1 deficiency contribute to OA progression. Here, we assumed that Enpp1 deficiency aggravates the chondrocyte apoptosis by regulating AMPK signaling pathway during the progress of OA.

## Materials and methods

### Experimental animals

**Fig. 1:**
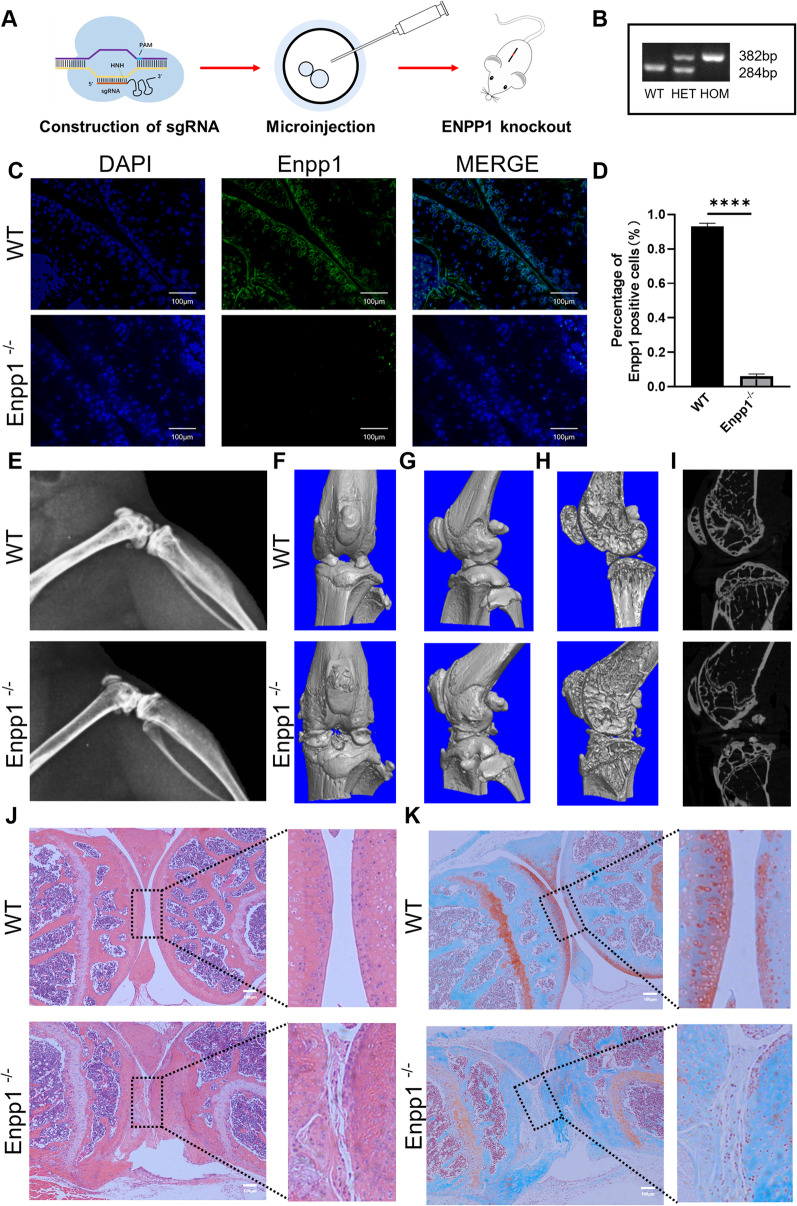
Enpp1 deletion caused knee OA: **A** Schematic for the construction of ENPP1 knockout (Enpp1^−/−^) mice using CRISPR/Cas9 technology. **B** The results of genotyping. **C** IF staining of Enpp1 in murine knee joint. (Enpp1, green; DAPI, blue; Scale bar: 100 µm, *n* ≥ 3). **D** The quantification of the expression of Enpp1 in WT and Enpp1^−/−^ knee joint (WT groups = 93.10% ± 1.90%, Enpp1^−/−^ groups = 6.03 ± 1.32, *n* = 3, *p* < 0.0001). **E** Representative lateral X-ray of WT and Enpp1^−/−^ knee joints at week 14 (*n* ≥ 3). **F**–**I** Representative micro-CT scan of WT and Enpp1^−/−^ knee joints at week 14. **J** H&E staining of WT and Enpp1^−/−^ knee joint at week 14 (Scale bar: 100 µm, *n* ≥ 3). **K** Safranin O-Fast Green staining of WT and Enpp1^−/−^ knee joints at week 14 (articular cartilage, red; bone, green; Scale bar: 100 µm, *n* ≥ 3).WT, wide type; HET: heterozygote; HOM homozygote; Enpp1, ectonucleotide pyrophosphatase/phosphodiesterase 1; IF, Immunofluorescence; DAPI, 4′,6-diamidino-2-phenylindole; WT, wild type; H&E, Hematoxylin & Eosin

All mouse experiments were approved by the ethical review board of the Shanghai East Hospital of Tongji University (Ethical approval code: EC. D (BG). 016.02.1). In this project, we use CRISPR/Cas9 technology to create stable Enpp1^−/−^ mice (Fig. 1A) and the relevant sequences were listed in Additional file 1. Considering the effect of estrogen on OA and the reproductive function of female mice [16], only male mice were used in the experiments.

### Genotyping

Genotyping was performed using toe genomic DNA extracted by small amount alkaline lysis. The mixed PCR primers of Enpp1 consists of tF1 (GATCCTGAGAGATTGGTACAGTCCAG), tR1 (CACAGAAATTGCCTGTGGTTTGC) and tR2 (TTGGCGCAGCTTGGTTTCAAC). Bands from agarose gel electrophoresis are 382 bp (WT mice) and 267 bp (Enpp1^−/−^ mice), respectively (Fig. 1B).

### Immunofluorescent staining and histological analysis of the knee joints

The knee joints of WT and Enpp1^−/−^ mice were harvested and stored in 10% formalin. Then knee joints were decalcified in 0.5 M EDTA (pH 7.4) for 4 weeks at 4 °C, changing EDTA every 2 days. The joints were made into 6-μm sections after embedding with paraffin. After deparaffinizing, the sections were incubated with proteinase K (20 μg/mL, Zymo Research, USA) for 10 min at room temperature and blocked in 5% normal serum (10,000 C, Thermo Fisher Scientific, USA) in PBS-T (0.4% Triton X-100 in PBS). Subsequently, the sections were incubated with antibodies against Enpp1 (1:1000, Bioss Antibodies Inc, Woburn, MA, USA) for 12 h and secondary antibody (Alexa Fluor 488 Labeled Goat Anti-Rabbit IgG, Beyotime, China) for an hour, at 37 ℃. Finally, the cover glass was placed on the section after adding the mounting medium. The images were captured by an inverted fluorescence microscope (Leica) and then the proportion of Enpp1-positive chondrocytes (green fluorescence) was analyzed by ImageJ.

The 6-μm sections described previously were stained with Hematoxylin & Eosin and Safranin O/Fast Green according to standard procedures.

### X-ray and micro-CT scan

Left hindlimbs of 14-week-old mice were harvested. X-rays of knee joints were taken with a Faxitron X-ray machine (Edimex) to evaluate the integrity and calcification of the knee surface. Besides, the knee joints were also scanned with 2 K resolution, 10-μm voxel size, 0.5 Al filter at 60 kV, and 167 μA. Images were reconstructed using NRecon version 1.1.11 (Bruker micro-CT) and analyzed using CTAn, v1.15 (SkyScan1176 in vivo micro-CT; Bruker).

### Isolation and cultivation of murine primary chondrocyte

WT and Enpp1^−/−^ mice were euthanized at postnatal D7. The articular cartilage from femoral condyles and tibia plateaus were incubated with collagenase type II (Gibco ™, Invitrogen Corporation, France) for two hours at 37℃. Cells were filtered through a 40-µm cell strainer and then cultured in DMEM culture media with 20% FBS. Chondrocytes after passage were cultured with 10% FBS. To test the effect of AICAR, WT and Enpp1^−/−^ chondrocytes were treated with AICAR (1 mM, MedChemExpress, New Jersey, United States) for 24 h.

### In vitro primary chondrocytes differentiation assay

Briefly, the glass slides were put into individual wells of 24-well plates and micromasses were cultured by pipetting 20 μL of primary chondrocytes on the glass slide for 4 h. To induce the chondrogenic differentiation, the micromasses were cultured in 10% FBS supplemented with Insulin-Transferrin-Selenium (ITS, zqxzbio, China) for 3 weeks and the medium was changed every three days. To analyze the chondrocytes differentiation, alcian blue staining of primary chondrocytes after inducing was performed. Quantificationally, the micromasses of chondrocytes after alcian blue staining were extracted with 150 μL of 6 M guanidine-HCl for 2 h at room temperature. Finally, the extracted dye was transferred to 96-well plates and the optical density measured at 620 nm [17].

### EDU cell proliferation assay and TUNEL (TdT-mediated dUTP Nick end labeling) staining

The proliferative ability of primary chondrocytes from WT and Enpp1^−/−^ mice was detected by EDU staining. Briefly, primary chondrocytes were equably planted in cell culture plates at approximately 50% density, then labeled and stained by using the Click-iT educ-488 cell proliferation assay kit (Servicebio, Wuhan, China).

The 6-μm knee sections (WT and Enpp1^−/−^ mice) and primary chondrocytes described previously were labeled and stained by TMR (red) TUNEL Cell Apoptosis Detection Kit (Servicebio, Wuhan, China).

The images (EDU and TUNEL staining) were captured by an inverted fluorescence microscope (Leica), and then the proportion of EDU-positive (green fluorescence) and TUNEL-positive (red fluorescence) chondrocytes was analyzed by ImageJ.

### High-throughput quantitative molecular measurement

Four male WT mice and four Enpp1^−/−^ mice at 14 weeks were euthanized, and their knee joints were separated. Then, the fresh samples of knee joints were sent to conduct Proteomic analysis using Nano-UPLCMSE tandem mass spectrometry. Bioinformatic analysis of proteins was performed by volcano plot, heatmap, KEGG pathway analysis, bar graph of enriched pathways, network of enriched pathways and protein–protein interaction networks.

### Western blotting

Primary chondrocytes from WT and Enpp1^−/−^ mice were split by RIPA lysis buffer (100 μL every 6-well plate) containing protease and phosphatase inhibitors (1:100). The adhesive cells were scraped down sufficiently using a brush, and the whole process was on the ice for 30 min. The cracked sample and 5 × protein loading buffer were added to the EP tube and boiled at 100 °C for 20 min.

Equal quantities (10 µL per lane) of proteins described above were performed by SDS–polyacrylamide electrophoresis, transferred onto PVDF membranes, and incubated with primary antibodies against AMPK (1:1000; Cell Signaling Technology, Boston, USA) and P-AMPK (1:1000; Cell Signaling Technology, Boston, USA). Then, membranes were incubated in secondary antibody at room temperature for 4 h (1:1000; Abclonal, Wuhan, China). Visualization of protein expression was captured using the Image Quant™ LAS 4000 imager (Fujifilm, Tokyo, Japan). Levels of protein expression were normalized with glyceraldehyde-3-phosphate dehydrogenase (GAPDH) expression and were analyzed quantitatively by ImageJ.

### Statistical analysis

All quantified data (at least three independent experimental groups) were described as mean ± standard deviation (SD). Two-tailed Student’s t test was used to compare the data of the two groups, and one-way analysis of variance (ANOVA) was used to compare the data more than two groups. Data analyses were performed using GraphPad Prism 6.02 (GraphPad Software Inc, San Diego, CA, USA). Statistical significance was determined at level of at least *p* ≤ 0.05 (*p < 0.05, **p < 0.01, ***p < 0.001, ****p < 0.0001). The signal of immunohistochemistry, EDU cell proliferation assay and TUNEL staining was calculated as the number of positive cells/visual field of view.

## Results

### Enpp1 deletion caused OA-like morphological changes of knee joints

To detect the efficiency of Enpp1 deletion, we examined the expression of Enpp1 in knee joints of WT (14 weeks) and Enpp1^−/−^ mice (14 weeks). As shown in Fig. 1C and Fig. 1D, the Enpp1 expression of chondrocytes membrane in Enpp1^−/−^ mice was dramatically decreased compared to WT mice. As the typical OA-like bone phenotypes of Enpp1^−/−^ mice from 8 to 22 weeks has been described by Jessica Bertrand et al. [13], we used X-ray and micro-CT to evaluate the changes. Compared to WT mice, discontinuity of articular cartilage surface, calcified articular cartilage and narrow joint space were found in X-ray of Enpp1^−/−^ mice knee joints at week 14 (Fig. 1E). The results of micro-CT revealed similar morphological changes (Fig. 1F–I). Interestingly, we also found significant osteoporosis in distal femur and proximal tibia thereby X-ray and micro-CT (Fig. 1E–I). To further analyze knee OA severity caused by Enpp1 deletion, H&E and Safranin O-Fast Green staining was performed. In Enpp1^−/−^ knee joints, we found a severe total erosion of cartilage compared to WT knee joints, which was revealed by the breakdown of the tidemark, surfaced regularity, turbulence of subchondral bone (Fig. 1J–K) and nearly vanished safranin O staining in Cartilage surface of knee joint (Fig. 1K). The above changes are highly similar to the pathological changes of knee OA.

### Enpp1 deletion in chondrocyte inhibits chondrocyte proliferation and chondrogenic differentiation

**Fig. 2:**
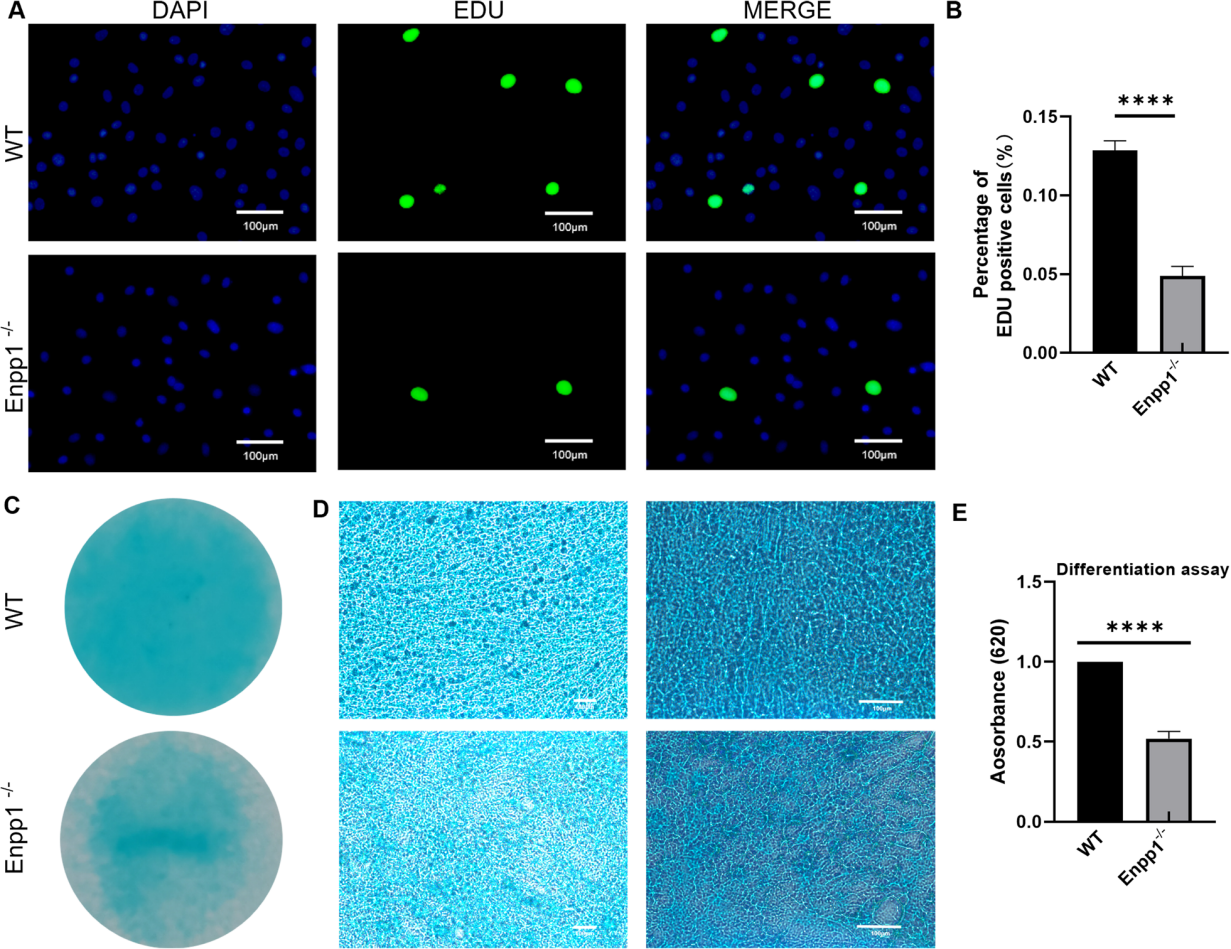
Enpp1 deletion impaired chondrocyte proliferation and chondrogenic differentiation: **A** EDU staining of WT and Enpp1^−/−^ primary chondrocyte (EDU-positive chondrocyte, green; DAPI, blue; scale bar: 100 µm, *n* ≥ 3). **B** The quantification of EDU-positive chondrocyte (WT groups = 12.867% ± 0.603% and Enpp1^−/−^ groups = 4.900% ± 0.600%, *p* < 0.0001, *n* ≥ 3). **C** Alcian blue staining of WT and Enpp1^−/−^ chondrocyte micromasses induced by 10% FBS with ITS for 21 days. **D** Microscope images of WT and Enpp1^−/−^ chondrocyte micromasses (left, 50 × , scale bar: 100 µm, *n* ≥ 3; right, 100 × , scale bar: 100 µm, *n* ≥ 3). **E** The quantitative analysis of Alcian blue staining by measuring the optical density of the micromasses extracted by guanidine-HCl from WT and Enpp1^−/−^ groups. Enpp1, ectonucleotide pyrophosphatase/phosphodiesterase 1; EDU, 5-Ethynyl-2′-deoxyuridine; WT, wild type; DAPI, 4′,6-diamidino-2-phenylindole; FBS, fetal bovine serum; ITS, Insulin-Transferrin-Selenium

To investigate whether Enpp1 deletion influence chondrocyte proliferation, chondrocytes were isolated from the articular cartilage of WT and Enpp1^−/−^ mice. We compared the proliferation ability of primary chondrocyte from WT and Enpp1^−/−^ mice by EDU staining. The results showed the proportion of newly proliferated chondrocytes in the control group was 2.6 times (12.87% and 4.90%) than that of Enpp1^−/−^ chondrocytes at the same time (Fig. 2A, B). Besides, to investigate the role of Enpp1 deletion in chondrogenic differentiation, the micromasses induced by 10% FBS with ITS were visualized thereby alcian blue staining and quantitative analysis. Visually and microscopically, more sulfated proteoglycan deposits were found in WT chondrocytes than Enpp1^−/−^ chondrocytes (Fig. 2C, D). Quantitatively, consistent with WT chondrocytes, sulfated proteoglycan deposits of Enpp1^−/−^ chondrocytes reduced by 48.03% (Fig. 2E). These results demonstrate that Enpp1 deletion impaired chondrocyte proliferation and chondrogenic differentiation.

### Enpp1 deletion in chondrocyte promotes articular cartilage apoptosis

**Fig. 3:**
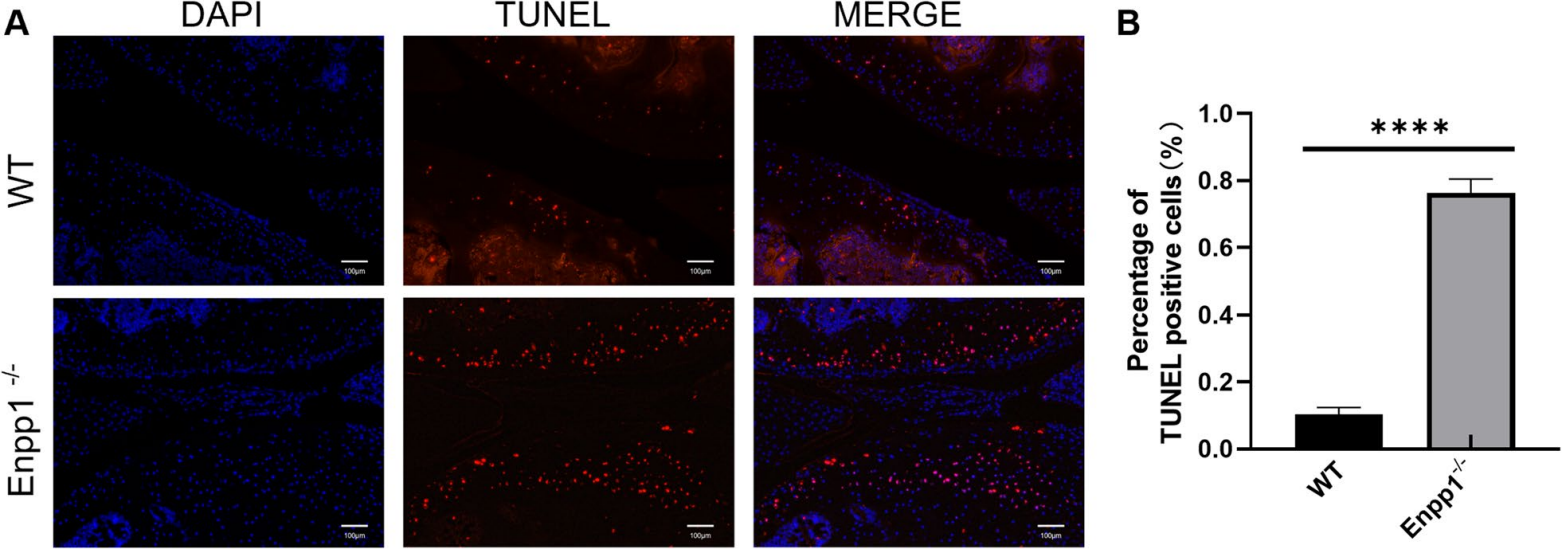
Loss of Enpp1 aggravates cell apoptosis in articular cartilage: **A** TUNEL staining of WT and Enpp1^−/−^ articular cartilage (TUNEL-positive chondrocyte, red; DAPI, blue; scale bar: 100 µm, *n* ≥ 3). **B** The percentage of TUNEL-positive chondrocytes in WT and Enpp1^−/−^ knee joints (WT groups = 10.33% ± 2.08% and Enpp1^−/−^ groups = 76.67% ± 3.79%, *p* < 0.0001, *n* ≥ 3)**.** Enpp1, ectonucleotide pyrophosphatase/phosphodiesterase 1; TUNEL, TdT-mediated dUTP-biotin nick end labeling; WT, wild type; DAPI, 4′,6-diamidino-2-phenylindole

To study whether Enpp1 deletion influence articular cartilage apoptosis, we used TUNEL staining to detect the apoptotic chondrocytes in WT and Enpp1^−/−^ joints (14 weeks). WT joints showed a dramatically higher proportion of TUNEL-positive cells than Enpp1^−/−^ joints (Fig. 3A). About 10.3% TUNEL-positive chondrocytes were detected in WT joints, while 76.3% in Enpp1^−/−^ joints (Fig. 3B). It is suggested that Enpp1 deletion seriously aggravated articular cartilage apoptosis.

### ATP metabolism, downregulated AMPK signaling pathway and chondrocyte apoptosis in the Enpp1^−/−^ articular cartilage may lead to knee OA-like phenotype

**Fig. 4:**
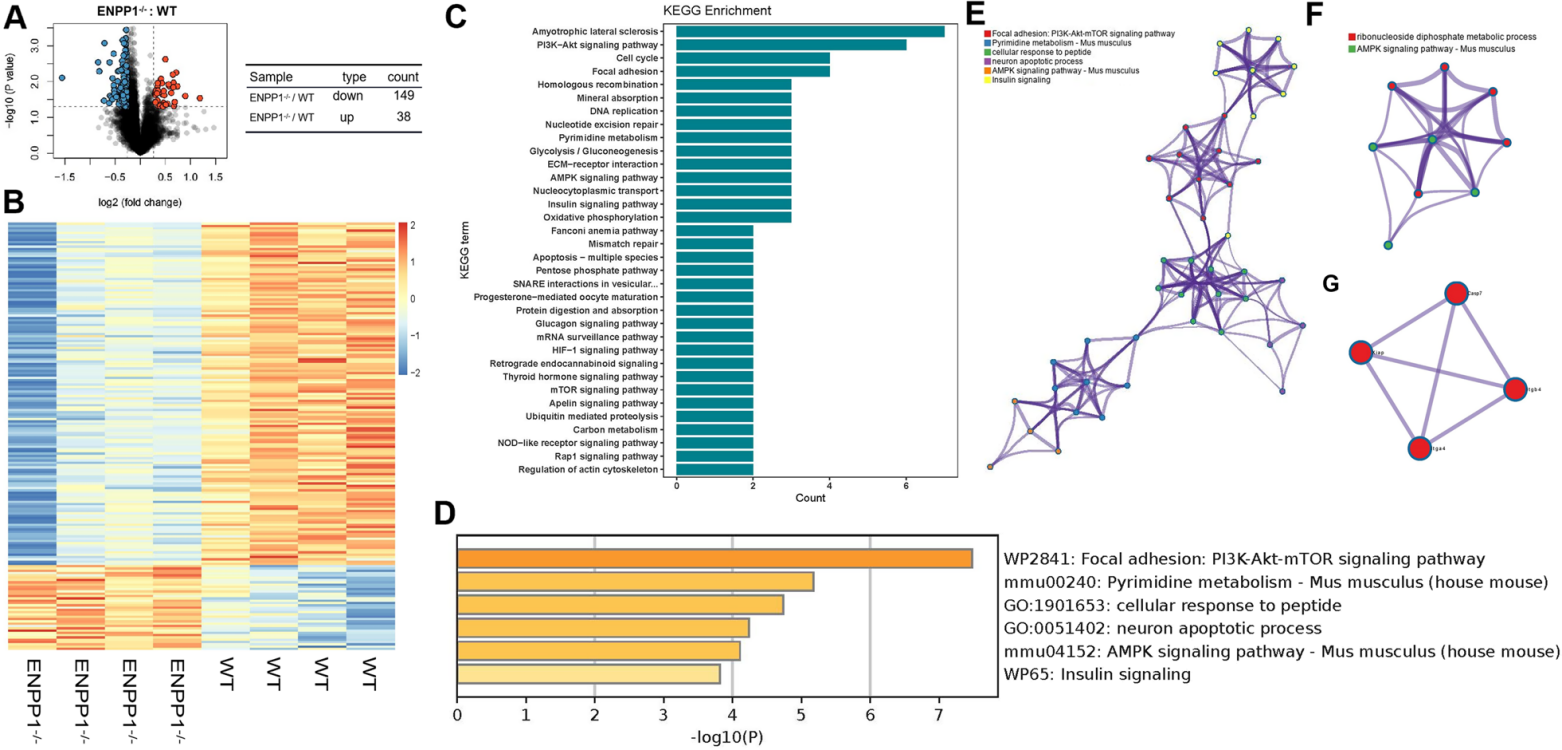
Enpp1 deletion caused unbalanced ATP metabolism, downregulated AMPK signaling pathway and chondrocyte apoptosis in knee articular cartilage: **A** Volcano plot showed 149 downregulated differential proteins and 38 upregulated differential proteins (*p* < 0.05, *n* ≥ 4). **B** Heatmap of 187 differential expression proteins in WT and Enpp1^−/−^ cartilages (*p* < 0.05, *n* ≥ 4). **C** KEGG pathway analysis of differential expression proteins (*p* < 0.05, *n* ≥ 4). **D** Bar graph of enriched pathways (Pyrimidine metabolism, AMPK signaling pathway, Apoptosis-multiple species, PI3K-Akt signaling pathway and mTOR signaling pathway) selected from Fig. C (*p* < 0.05, *n* ≥ 4). **E** Network of enriched pathways (pyrimidine metabolism, AMPK signaling pathway, Apoptosis-multiple species, PI3K-Akt signaling pathway and mTOR signaling pathway) chosen from Fig. C (*p* < 0.05, *n* ≥ 4). **F** Bar graph of enriched pathways (pyrimidine metabolism and AMPK signaling pathway) (*p* < 0.05, *n* ≥ 4). **G** Protein–protein interaction network diagram of key modules was related to Casp7, Xiap, Itga4 and Itgb4. Enpp1, ectonucleotide pyrophosphatase/phosphodiesterase 1; ATP, Adenosine triphosphate; AMPK, AMP-activated protein kinase; WT, wild type; KEGG, Kyoto Encyclopedia of Genes and Genomes; PI3K, Phosphoinositide 3-Kinase; Akt, protein kinase B; mTOR, mammalian target of rapamycin; Casp7, caspase 7; Xiap, X-linked inhibitor-of-apoptosis protein; Itga4, integrin a4; Itgb4, integrin beta4

To find out the differentially expressed proteins and signaling pathways in articular cartilage following Enpp1 deletion, the cartilages of the proximal tibia and distal femur were analyzed by High-throughput protein sequencing. Comparing the protein expression between WT and Enpp1^−/−^ cartilages, 187 differentially expressed proteins (upregulate: 38; downregulate:149;) were found (Fig. 4A, all *p* < 0.05) and used to create a heatmap (Fig. 4B, p < 0.005). We used KEGG pathway analysis to classify differential proteins and 34 differentially expressed signaling pathways were screened out (Fig. 4C). Given that knee OA is highly correlated with ATP metabolism, AMPK signaling pathway, and chondrocyte apoptosis, we analyzed the relevant signaling pathways (Fig. 4D) and protein interactions (Fig. 4E–G) among 34 differentially expressed signaling pathways. In Fig. 4G, protein–protein interaction network diagram of key modules was related to Casp7, Xiap, Itga4 and Itgb4 and above-mentioned proteins is highly associated with cell apoptosis [18–21]. As Enpp1 mainly catalyzes ATP to AMP, which activates AMP signaling pathway, we assumed that Enpp1 deletion induces chondrocyte apoptosis by inhibiting AMPK signaling pathway and thus leads to the progress of knee OA.

### Enpp1 deficiency caused chondrocyte apoptosis by inhibiting AMPK signaling pathway

**Fig. 5:**
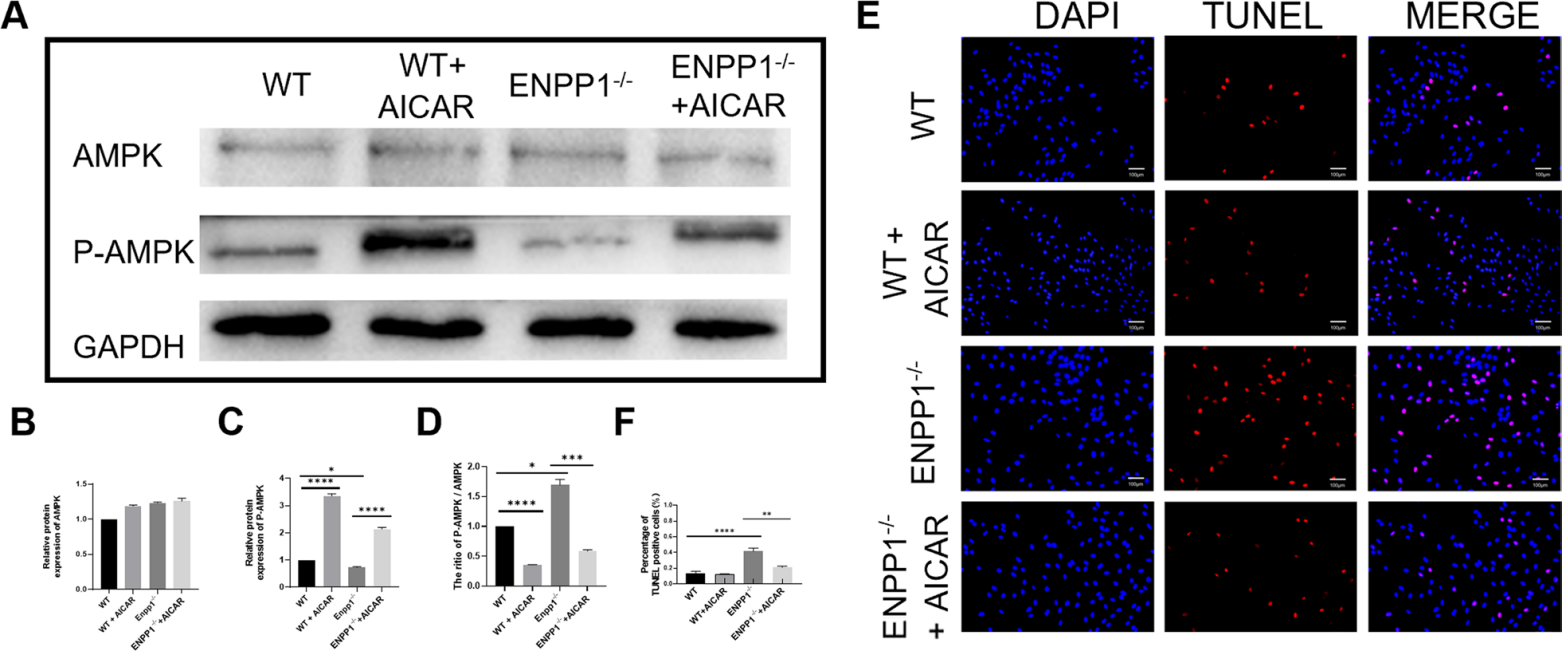
AICAR can reverse the inhibition of AMPK phosphorylation due to Enpp1 deficiency: **A** Western blot of chondrocytes (WT, WT + AICAR, Enpp1^−/−^, Enpp1^−/−^ + AICAR, respectively; *p* < 0.05, *n* ≥ 3)); **B**–**D** quantitative statistic of Western blot (*p* < 0.05, *n* ≥ 3); **E** TUNEL staining of chondrocytes (WT, WT + AICAR, Enpp1^−/−^ and Enpp1^−/−^ + AICAR, respectively; *p* < 0.05, *n* ≥ 3); **F** Quantitative analysis of TUNEL-positive chondrocytes (*p* < 0.05, *n* ≥ 3). AICAR, Acadesine; AMPK, AMP-activated protein kinase; Enpp1, ectonucleotide pyrophosphatase/phosphodiesterase 1; WT, wild type; TUNEL, TdT-mediated dUTP-biotin nick end labeling; DAPI, 4′,6-diamidino-2-phenylindole

To identify the relationship between Enpp1 deficiency and AMPK signaling pathway, we detected the expression of AMPK signaling pathway by western blot. The results of Fig. 5A–D demonstrated that AMPK signaling pathway (the phosphorylation process of AMPK) was significantly inhibited by Enpp1 deficiency and this change can be reversed by AICAR. This is consistent with the results of high-throughput protein sequencing. To further verify whether AICAR can save the chondrocyte apoptosis, Enpp1^−/−^ chondrocyte cultivated with AICAR was performed by TUNEL staining. Contrast with WT chondrocyte, Enpp1 deficiency increased the proportion of TUNEL-positive cells (from 13.7 to 41.5%) and apoptotic phenotype can be rescued by AICAR (from 41.5 to 21.1%). No significant differences were found of TUNEL-positive cells between WT and WT + AICAR group (Fig. 5E, F). These observations indicated that Enpp1 deficiency caused chondrocyte apoptosis by inhibiting AMPK signaling pathway.

## Discussion

Articular cartilage degeneration, the main cause of knee OA, is characterized by decreased cartilage proliferation, damaged chondrocyte differentiation and increased cartilage apoptosis. We have shown that downregulated AMPK signaling pathway contributes to the articular cartilage apoptosis of Enpp1^−/−^ knee joints. Besides, we found Enpp1 deficiency also influenced the chondrocyte proliferation and chondrogenic differentiation.

Enpp1 is crucial for the ATP hydrolysis in osteochondral development [22]. It is well known that tissue mineralization is mainly determined by the concentration of hydroxyapatite, which is mainly influenced by the ratio of calcium (Ca^2+^) and phosphate (Pi). PPi has a dual identity that PPi can not only inhibit the production of hydroxyapatite, but also be decomposed into Pi, the promoter of hydroxyapatite. Thus, during the reaction of Enpp1 hydrolyzes ATP into AMP and PPi outside the cell, Enpp1 deficiency decreases AMP/ ATP and PPi/ Pi. Roberts et al. [23] thought Enpp1 is essential for the regulation of osteochondral development because of the PPi generated from ATP metabolism.

Pathologic changes of Enpp1 mutation-associated diseases are mainly due to abnormal PPi and AMP levels. Enpp1 knockout mice display a severe calcification phenotype, with reduced levels of extracellular PPi and AMP and the pathological calcification is most commonly involved in soft tissues, such as arterial walls [14, 15]. Generalized arterial calcification of infancy (GACI), a widely calcified syndrome of the whole body caused by inactivating mutations in Enpp1, is charactered by reduced plasma PPi [24] (virtually no PPi in GACI patients’ blood) and AMP [25]. As reduced PPi and AMP concentrations of GACI patients, its preferred treatment must be PPi and AMP supplementation perpetually. Researches showed that orally administered PPi is not effective in inhibiting tissue calcification of GACI patients [26] because PPi is eliminated by phosphatases in the gut [27]. Yvonne Nitschke et al. [28] tried to treat arterial stenoses, occurring in at least 72.4% GACI cases caused by Enpp1 deficiency. They found the addition of neither PPi nor etidronate, a current off-label treatment for GACI, had no effect on proliferation capacity of vascular smooth muscle cell (VSMC), but supplementation of recombinant humanEnpp1-Fc protein (rhEnpp1-Fc), AMP or adenosine restored the silenced Enpp1-associated proliferation.

The reduced PPi and AMP levels contribute to OA-like changes caused by Enpp1 deficiency. Enpp1 knockout mice display a tiptoe-walking phenotype, with serious cartilage calcification [26]. Increasing evidence revealed the relationship between Enpp1 and abnormal PPi/ Pi in the progress of OA [29, 30], and multiple differentially expressed signaling pathways were involved in Enpp1-associated OA. Jin et al. [12] discovered metacarpophalangeal osteoarthritis of Enpp1^−/−^ mice, and they explained the mechanism clearly. In their study, the role of Hedgehog (Hh) signaling pathway was selected and validated as the primary mechanism for articular calcification and OA due to Enpp1 deficiency. Besides, Jessica Bertrand et al. [13] reported that cartilage calcification in Enpp1 null mice results in increased extracellular matrix (ECM) calcification and activation of canonical Wnt signaling pathway. Moreover, the osteochondral phenotypes in 10-week-old Enpp1^−/−^ mice are driven by catalytically independent Enpp1 function, while the osteochondral changes in 23-week-old Enpp1^−/−^ mice ascribe to low plasma PPi [31]. All of the above studies attributed OA-like phenotypes of Enpp1^−/−^ mice mainly to PPi/ Pi abnormalities.

Low AMP/ATP resulted from Enpp1 deficiency causes OA by inhibiting AMPK signaling pathway. Low AMP/ATP in plasma is widely reported in Enpp1-deficient humans and mice [24–27]. It is widely known that AMPK, a metabolic checkpoint, is activated in response to an increased AMP/ATP ratio [32, 33]. AMPK activity in chondrocytes is essential in keeping joint homeostasis and OA progress. Phosphorylation of AMPK is decreased in human OA chondrocytes [34], and suppressing AMPK signaling pathway can lead to OA progression [35]. Besides, the chondrocyte apoptosis is significantly upregulated after the conditional deletion of AMPK in chondrocytes [36], which is highly consistent with our results of Figs. 4 and 5. Combining the results of proteomic analysis (Fig. 4), the function of Enpp1 and OA, we screened out the differentially expressed signaling pathways related to cartilage metabolism (Pyrimidine metabolism, Glycolysis/Gluconeogenesis, AMPK signaling pathway, Pentose phosphate pathway and Glucagon signaling pathway) and chondrocyte apoptosis (PI3K-Akt signaling pathway, AMPK signaling pathway and mTOR signaling pathway) between WT and Enpp1^−/−^ articular cartilages. Therefore, we suggested that Enpp1 deficiency may mediate chondrocytes apoptosis through AMPK signaling pathway which is highly correlated ATP metabolism and consequently cause OA. Figure 5 verifies this hypothesis. We found that AICAR, an adenosine analog which activates AMPK signaling pathway, can save the inhibition of AMPK phosphorylation (Fig. 5A) and chondrocyte apoptosis induced by Enpp1 deficiency. Therefore, AMPK signaling pathway plays a key role in OA of Enpp1 deficiency.

**Fig. 6:**
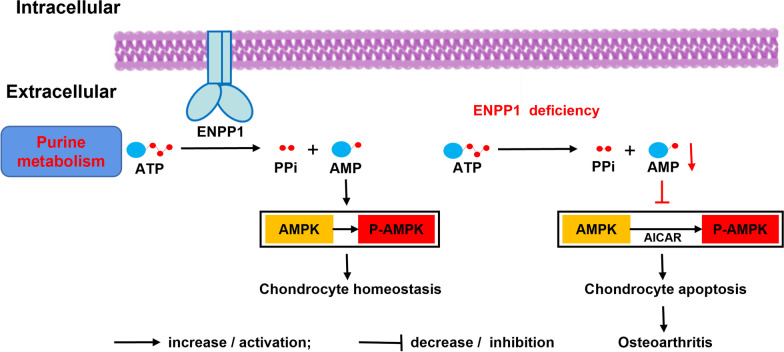
Diagram of proposed mechanism of Enpp1 deficiency caused chondrocyte apoptosis. Enpp1: ectonucleotide pyrophosphatase/phosphodiesterase 1; ATP: adenosine triphosphate; AMP: adenosine monophosphate; PPi: pyrophosphate; AMPK: AMP-activated protein kinase; P-AMPK: phosphorylation of AMPK; AICAR: Acadesine

Interestingly, Enpp1 deficiency can lead to osteoporosis and articular cartilage calcification, two antipodal fates. In our previous research, we found that Enpp1 deficiency causes mouse osteoporosis via the MKK3/p38 MAPK/PCNA signaling pathway [37]. In the progression of osteoporosis, disorders of energy metabolism availability cause osteoblast dysfunction and lead to inhibition of bone formation [38]. Inversely, in the process of cartilage calcification, abnormal chondrocyte metabolism causes increased osteogenic differentiation ability and decreased chondroblast differentiation ability [12]. It can be summarized as that cell metabolism maintains programmed phenotype and pathological factors of disrupting cell metabolism, such as Enpp1 deficiency, can cause adverse cell fates (Fig. 6).

Collectively, our findings demonstrated that Enpp1 plays an indispensable role in chondrocyte metabolism through AMPK signaling pathway. Enpp1 deficiency in chondrocyte increases chondrocyte apoptosis, inhibits chondrocyte proliferation, impairs chondrogenic differentiation, and interrupts the activation of AMPK signaling pathway (phosphorylation of AMPK). Till now, it is still not clear what is the detail mechanism of chondrocyte metabolism regulated by Enpp1. The mechanism of chondrocyte proliferation and chondrogenic differentiation affected by Enpp1 also remains unclear. We are still trying to uncover the aforementioned mechanisms that are not fully understood.

## Supplementary Information

**Additional file 1.** The construction strategy of Enpp1^-/-^ mice.

## Data Availability

Authors can confirm that all relevant data are included in the article or its supplementary information files.

## Abbreviations

Enpp1: Ectonucleotide pyrophosphatase/phosphodiesterase 1
OA: Osteoarthritis
WT: Wild-type
AMPK: AMP-activated protein kinase
P-AMPK: Phosphorylation of AMPK
AICAR: Acadesine
ATP: Adenosine triphosphate
AMP: Adenosine monophosphate
PPi: Pyrophosphate
Pi: Pyrophosphate
ECM: Extracellular matrix
Wnt: Wingless/Integrated

## References

[1] Cross M, Smith E, Hoy D, Nolte S, Ackerman I, Fransen M (2014). The global burden of hip and knee osteoarthritis: estimates from the global burden of disease 2010 study. Ann Rheum Dis.

[2] Clarke J (2020). Knee OA increases risk of all-cause mortality. Nat Rev Rheumatol.

[3] Tan L, Register TC, Yammani RR (2020). Age-related decline in expression of molecular chaperones induces endoplasmic reticulum stress and chondrocyte apoptosis in articular cartilage. Aging Dis.

[4] Lin S, Li H, Wu B, Shang J, Jiang N, Peng R (2022). TGF-β1 regulates chondrocyte proliferation and extracellular matrix synthesis via circPhf21a-Vegfa axis in osteoarthritis. Cell Commun Signal.

[5] Hou M, Zhang Y, Zhou X, Liu T, Yang H, Chen X (2021). Kartogenin prevents cartilage degradation and alleviates osteoarthritis progression in mice via the miR-146a/NRF2 axis. Cell Death Dis.

[6] Evans WH, Hood DO, Gurd JW (1973). Purification and properties of a mouse liver plasma-membrane glycoprotein hydrolysing nucleotide pyrophosphate and phosphodiester bonds. Biochem J.

[7] Onyedibe KI, Wang M, Sintim HO (2019). ENPP1, an old enzyme with new functions, and small molecule inhibitors-A STING in the tale of ENPP1. Molecules.

[8] Miyoshi N, Oubrahim H, Chock PB, Stadtman ER (2006). Age-dependent cell death and the role of ATP in hydrogen peroxide-induced apoptosis and necrosis. Proc Natl Acad Sci U S A.

[9] Zhang J, Wang Y, Chen J, Liang X, Han H, Yang Y (2017). Inhibition of cell proliferation through an ATP-responsive co-delivery system of doxorubicin and Bcl-2 siRNA. Int J Nanomedicine.

[10] Iwamoto T, Nakamura T, Doyle A, Ishikawa M, de Vega S, Fukumoto S (2010). Pannexin 3 regulates intracellular ATP/cAMP levels and promotes chondrocyte differentiation. J Biol Chem.

[11] Stefan C, Jansen S, Bollen M (2005). NPP-type ectophosphodiesterases: unity in diversity. Trends Biochem Sci.

[12] Jin Y, Cong Q, Gvozdenovic-Jeremic J, Hu J, Zhang Y, Terkeltaub R (2018). Enpp1 inhibits ectopic joint calcification and maintains articular chondrocytes by repressing hedgehog signaling. Development.

[13] Bertrand J, Kräft T, Gronau T, Sherwood J, Rutsch F, Lioté F (2020). BCP crystals promote chondrocyte hypertrophic differentiation in OA cartilage by sequestering Wnt3a. Ann Rheum Dis.

[14] Zheng L, Zhang Z, Sheng P, Mobasheri A (2021). The role of metabolism in chondrocyte dysfunction and the progression of osteoarthritis. Ageing Res Rev.

[15] Hwang HS, Kim HA (2015). Chondrocyte apoptosis in the pathogenesis of osteoarthritis. Int J Mol Sci.

[16] Sniekers YH, Weinans H, Bierma-Zeinstra SM, van Leeuwen JP, van Osch GJ (2008). Animal models for osteoarthritis: the effect of ovariectomy and estrogen treatment - a systematic approach. Osteoarthr Cartil.

[17] Liu M, Alharbi M, Graves D, Yang S (2020). IFT80 is required for fracture healing through controlling the regulation of TGF-β signaling in chondrocyte differentiation and function. J Bone Miner Res.

[18] Ding Y, Lang Y, Zhang H, Li Y, Liu X, Li M (2022). Candesartan reduces neuronal apoptosis caused by ischemic stroke via regulating the FFAR1/ITGA4 pathway. Mediat Inflamm.

[19] Carter BZ, Mak DH, Schober WD, Koller E, Pinilla C, Vassilev LT (2010). Simultaneous activation of p53 and inhibition of XIAP enhance the activation of apoptosis signaling pathways in AML. Blood.

[20] Han L, Wang L, Tang S, Yuan L, Wu S, Du X (2018). ITGB4 deficiency in bronchial epithelial cells directs airway inflammation and bipolar disorder-related behavior. J Neuroinflamm.

[21] Lin YF, Lai TC, Chang CK, Chen CL, Huang MS, Yang CJ (2013). Targeting the XIAP/caspase-7 complex selectively kills caspase-3-deficient malignancies. J Clin Invest.

[22] Mackenzie NC, Huesa C, Rutsch F, MacRae VE (2012). New insights into NPP1 function: lessons from clinical and animal studies. Bone.

[23] Roberts F, Zhu D, Farquharson C, Macrae VE (2019). ENPP1 in the regulation of mineralization and beyond. Trends Biochem Sci.

[24] Rutsch F, Ruf N, Vaingankar S, Toliat MR, Suk A, Höhne W (2003). Mutations in ENPP1 are associated with 'idiopathic' infantile arterial calcification. Nat Genet.

[25] Dedinszki D, Szeri F, Kozák E, Pomozi V, Tőkési N, Mezei TR (2017). Oral administration of pyrophosphate inhibits connective tissue calcification. EMBO Mol Med.

[26] Orriss IR, Arnett TR, Russell RG (2016). Pyrophosphate: a key inhibitor of mineralisation. Curr Opin Pharmacol.

[27] Ferguson A, Watson WC, Maxwell JD, Fell GS (1968). Alkaline phosphatase levels in normal and diseased small bowel. Gut.

[28] Nitschke Y, Yan Y, Buers I, Kintziger K, Askew K, Rutsch F (2018). ENPP1-Fc prevents neointima formation in generalized arterial calcification of infancy through the generation of AMP. Exp Mol Med.

[29] Ramaswamy J, Nam HK, Ramaraju H, Hatch NE, Kohn DH (2015). Inhibition of osteoblast mineralization by phosphorylated phage-derived apatite-specific peptide. Biomaterials.

[30] Danino O, Svetitsky S, Kenigsberg S, Levin A, Journo S, Gold A (2018). Inhibition of nucleotide pyrophosphatase/phosphodiesterase 1: implications for developing a calcium pyrophosphate deposition disease modifying drug. Rheumatology.

[31] Maulding ND, Kavanagh D, Zimmerman K, Coppola G, Carpenter TO, Jue NK (2021). Genetic pathways disrupted by ENPP1 deficiency provide insight into mechanisms of osteoporosis, osteomalacia, and paradoxical mineralization. Bone.

[32] Cairns RA, Harris IS, Mak TW (2011). Regulation of cancer cell metabolism. Nat Rev Cancer.

[33] June RK, Liu-Bryan R, Long F, Griffin TM (2016). Emerging role of metabolic signaling in synovial joint remodeling and osteoarthritis. J Orthop Res.

[34] Terkeltaub R, Yang B, Lotz M, Liu-Bryan R (2011). Chondrocyte AMP-activated protein kinase activity suppresses matrix degradation responses to proinflammatory cytokines interleukin-1β and tumor necrosis factor α. Arthritis Rheum.

[35] Petursson F, Husa M, June R, Lotz M, Terkeltaub R, Liu-Bryan R (2013). Linked decreases in liver kinase B1 and AMP-activated protein kinase activity modulate matrix catabolic responses to biomechanical injury in chondrocytes. Arthritis Res Ther.

[36] Zhou S, Lu W, Chen L, Ge Q, Chen D, Xu Z (2017). AMPK deficiency in chondrocytes accelerated the progression of instability-induced and ageing-associated osteoarthritis in adult mice. Sci Rep.

[37] Wang Q, Gao Z, Guo K, Lu J, Wang F, Huang Y (2022). ENPP1 deletion causes mouse osteoporosis via the MKK3/p38 MAPK/PCNA signaling pathway. J Orthop Surg Res.

[38] Lee WC, Guntur AR, Long F, Rosen CJ (2017). Energy metabolism of the osteoblast: implications for osteoporosis. Endocr Rev.

