# Supplementary material for: Enpp1 deficiency caused chondrocyte apoptosis by inhibiting AMPK signaling pathway
Source: J Orthop Surg Res. 2023 Jun 27;18:462. doi: 10.1186/s13018-023-03923-1 (PMC10294376; doi:10.1186/s13018-023-03923-1)
Supplement: Supplementary file 1 — Additional file 1. The construction strategy of Enpp1-/- mice. [file 13018_2023_3923_MOESM1_ESM.doc]

**The construction strategy of Enpp1^-/-^ mice by using CRISPR/Cas9.**

**1. gRNA Sequence**

gRNA1 Sequence（5’ - 3’）: ACCCTCAGATGTGCCATGCA

gRNA1 Sequence（5’ - 3’）: TGCTTTAGCAACCAACTACT

**2. Wildtype Genomic Sequence**

gRNAs are marked in blue, exons are marked in red

CGCACG CAGTGG GGGGGG GGTGTT GTTCTG TGGATT ATTTTC CTCCTT TGTCTT CTGACC CTCAAA ACTTGT TGTTGA CCTTAG GGGTTC CAAGCT CAGTCA TTTTAA ATATTA AAGGCA GGAATC ACGGGG AGGGAG AAGGTG AGAAAA CAGAAA CCCTGG AAGGAT CCTGAG AGATTG GTACAG TCCAGC TCTCTG CTTTTA CAGATG AATAAG CAGATG CTTAGA CCTTAT GAACCCCTTCAG CTCATA CTACCA GCTCAT GGCAGT GCTGTG GCCCAG TGGTCA TTCCCA TGCCCA **TGCATG GCACAT CTGAGG GT**GCTG TTCTGG GTTTAG GTTTGT GAGGTC ACGGCT AGCACG TTCCTA GATGGC AGTAAG AAGTGG AGTCTC AATAGA TTTCCT TCTTTT AAGGAT TACAAC TTTCTT CTGGCT AATTCC TTTGTG TGAGCC AAGTGT TACATT ATCTTT GGATGA TTTAGA AAGTTG CTGTCT GACGTT TATCAC CATCTC CAGAAC AGGACA TAAATC ACAAGT ATGTGG TCTGAA ATGTTT AAAAAA TGAGCC TGTCCC TGTGAT CAGCTG CCACAT CTGGAA ACACTG CAAAGT CAGAGA CATGGA AAGGGA TGAAGT TGGGGG AATAGG AGGATG TCAGAA GGCTCT AACAGT CTTTTT CTCTCC TTACAG **GTTTTG TCAGTA TGTGTG CTAACA ACAATT CTTGGT TGTATA TTTGGG TTGAAA CCAAGC TGCGCC AAAGAA G**GTAAT TAATGT TGTGGG GTTCTG TGTGTC TGTGCC TGTGGA TGCATG TGTGCC TATGTG CATCTC TGTGTG TATGTG TCTCTG TGTGTT TCTCTG TGTTTG CATATG TGTATG ATTACA TCTATA TGTATA TGTGCA TGTGTG TGTGTG TGTGTG TGTGTG TGTGTG TATTTG GCATTA TGATAT GATTGA GACAAC TGTAAT CTTCAA GTCCTG TGCATA CTCCAC AGATGT TGAAAT TTTCAT AAGTTT GATGCT GGTTTA AGCACT CTTCCT AAAGTC CTATCT TATTTT GGCCAT ACAAAG GGTCTT TTTTGC TTACAA ATTTTT ATATTA AAATGT GGAGTA GAGTCT GTGACC AAAAAA CAACTC TCCACC CCTAAA GAAAAA CCACGA AGACAT GACCAG AGGTTC CTCCAA AGCCCC A**AGTAG TTGGTT GCTAAA GCA**GCT CTGGAG GTTACT AGGAGA CCAGTT AGCATC CCTAAG TGACAG AGTCTC AAGAGA GTTTTT AAGATA TTTCTG CCATTG CTAATT TCAAAG TAGTAA TGAACT CATCAC ACTTCT AATTGT GTTTTC CTAGTA TCAAGA ACAGGC TCTATA TAACAC CAAAAA AAACAC CATATA GGTAAT GAAAGC AAACCA CAGGCA ATTTCT GTGGTC ATTCTT TTGATT CGGTAA TGTCTC
